# Gastrointestinal effects of *Nigella sativa* and its main constituent, thymoquinone: a review

**Published:** 2016

**Authors:** Farzaneh Shakeri, Zahra Gholamnezhad, Bruno Mégarbane, Ramin Rezaee, Mohammad Hosein Boskabady

**Affiliations:** 1 *Neurogenic Inflammation Research Centre and Department of Physiology, School of Medicine, Mashhad University of Medical Sciences, Mashhad, Iran*; 2 *Department of Medical and Toxicological Critical Care, Paris-Diderot University, INSERM U1144, Paris, France*; 3 *Department of Physiology and Pharmacology, School of Medicine, North Khorasan University of Medical Sciences, Bojnurd, Iran*

**Keywords:** *Nigella sativa*, *Gastrointestinal disease*, *Thymoquinone*

## Abstract

Gastrointestinal (GI) diseases affect a large number of people all over the world. Uncontrolled acid secretion and occurrence of gastric ulcers are common disorders of GI tract which pose serious problems to human health. Many synthetic drugs have been used to treat GI disorders but a definite cure has not been discovered so far and the available medications cause several side effects.

*Nigella sativa *(*N. sativa*) (Ranunculacea) has several therapeutic effects which are attributed to its constituents like nigellicine, nigellidine, thymoquinone, dithymoquinone, thymol and carvacrol. Several beneﬁcial pharmacological properties of this plant such as anti-oxidant, anti-bacterial, anti-histaminic, anti-hypertensive, hypoglycemic, anti-fungal, anti-inﬂammatory, anti-cancer and immunomodulatory effects were reported and different therapeutic properties such as reliving bronchial asthma, jaundice, hydrophobia, paralysis, conjunctivitis, piles, skin diseases, anorexia, headache, dysentery, infections, obesity, back pain, hypertension and gastrointestinal problems, have been described for the seeds of *N. sativa* and its oil.

The present review provides a detailed summery of scientific researches regarding gastrointestinal effect of *N. sativa* and its main constituent, thymoquinone.

## Introduction

Gastrointestinal diseases refer to conditions involving the esophagus, stomach, small intestine, large intestine, rectum, liver, gallbladder and pancreas. Symptoms of gastrointestinal disorders include pain, heartburn, dyspepsia, abdominal distension, nausea, vomiting, bloating, constipation and diarrhea. Among these, functional and motility disorders are the most common conditions seen in clinical practice and among the general population. The prevalence of these disorders in western countries is about 10-20%. Gastrointestinal diseases are chronic conditions which need long term medication and decrease the quality of life (Drossman, 1993). Commonly used drugs have several side effects like osteoporosis, disturbance in small intestine flora, kidney stones, anemia and increased chance of occurrence of drug-induced diseases such as gastric cancer. Therefore, due to the side effects of conventional medicine, the use of natural products in the treatment of various diseases has been in the center of attention in the last few decades.

Herbal medicine has been traditionally used for the treatment of different ailments.* Nigella sativa* (*N. sativa*) is used as an important drug in the traditional medicine like Unani and Ayurveda (Goreja, 2003 ▶; Sharma et al., 2005 ▶). Almost 80% of the populations of developing countries rely mainly on herbal medicine in primary medical therapy (Mantle et al., 2000 ▶). Medicinal plants are the source of enormous drugs and many important drugs were derived directly or indirectly from plants or from molecules of plant origin.

*N. sativa*, commonly known as black seed or black cumin, is an annual flowering plant from the family of Ranunculaceae, which is native to southern Europe, North Africa and Southwest Asia. *N. sativa* seeds include oil, protein, carbohydrate, fiber, saponin, moisture and the oil extracted from *N. sativa *is mostly consisted of linoleic acid, oleic, dihomolinoleic acid, palmitic acid stearic acid, myristic acid, stroles and eicodadienoic acid (El-Tahir and Bakeet, 2006 ▶).

 Based on the use of *N. sativa* in folk medicine as a natural remedy for a number of diseases, scientists have studied its effects on conditions such as asthma (Boskabady et al., 2010 ▶), hypertension (Dehkordi and Kamkhah, 2008 ▶), diabetes (Bamosa et al., 2010 ▶) and inflammation (Chehl et al., 2009 ▶). Moreover, this herb is known to have antioxidant (Burits and Bucar, 2000 ▶), analgesic and anti-pyretic (Al-Ghamdi, 2001 ▶), anti-schistosomiasis (Mohamed et al., 2005 ▶), anti-fungal (Islam et al., 1989 ▶), anti-bacterial (Morsi, 1999 ▶), anti-convulsant (Raza et al., 2008 ▶), anti-cancer (Mahmoud and Torchilin, 2013), hepatoprotective (Kanter et al., 2005a) and Neuroprotective activities (Khazdair, 2015 ▶). In addition, it showed healing potential in gastrointestinal disturbances (Al Mofleh et al., 2008 ▶).

In last three decades, numerous researches have been done to identify plant-derived natural substances and understand the mechanisms of their pharmacological actions. *N. sativa* extract increases the activity of antioxidant enzymes (catalase, glutathione peroxidase, and glutathione-s-transferase) and acts as a free radical scavenger. As an anti-cancer agent, its effects such as modulation of the activities of molecular targets including p53, p73, PTEN, STAT3, PPAR-g, activation of caspases, and generation of ROS have been demonstrated. It also suppresses inflammatory mediators, leukotrienes, prostaglandins, and B cell-mediated immune response while balances Th1/ Th2 responses and potentiates T cell and natural killer cell-mediated immune responses (Gholamnezhad et al., 2014 ▶), as an anti-inflammatory and immunomodulatory agent. In this regard, several studies demonstrated that *N. sativa *has anti-cancer, hepatoprotective, anti-bacterial, anti-schistosomiasis, anti-inflammatory and antioxidant activities in gastrointestinal system (Gholamnezhad et al., 2015 ▶).

 In this review, the gastrointestinal effects of *N. sativa *and thymoquinone (TQ) were reviewed.

## Methods

To collect the related data on gastrointestinal effects of *N. sativa *and its main constituent, TQ, online literature resources including Medline, Pubmed, Science Direct, Scopus, and Google Scholar websites was checked from 1989 to 2015.

**Gastrointestinal effects**

**Anti-cancer effect**

Colorectal cancers (development of cancer in the colon or rectum) start as a polyp – a growth that starts in the inner lining of the colon or rectum and progresses toward the center. The preventive effect of *N. sativa* oil on rat colon cancer induced by 1,2-dimethylhydrazine was investigated. The animals were divided into four groups: Group 1 served as control; Group 2 received oil at post-initiation stage; Group 3 received oil at the initiation stage and Group 4 received 0.9% saline and oil from the beginning until the end of the study. The results of this study showed that *N. sativa* oil significantly reduced the total number of aberrant crypt foci in the post-initiation stage (group 2) whereas it showed no significant inhibitory effect on initiation stage (group 3). The results indicated that *N. sativa* oil has potent preventive effect on colon carcinogenesis in the post-initiation stage (Salim and Fukushima, 2003 ▶).

The preventive effect of TQ, the main constituent of *N. sativa* on HCT-116 human colorectal cancer cells was evaluated. The results showed that TQ is a potent agent against colon cancer cells and triggers apoptosis via a p53-dependent mechanism (Gali-Muhtasib et al., 2004 ▶). On the other hand, another study revealed that TQ has no effect against HEp-2 cancer cells (Rooney and Ryan, 2005 ▶).

The effect of TQ on pancreatic cancer cells and mucin 4 (MUC4) expressions was evaluated. MUC4 is expressed in pancreatic cancer and it contributes to the regulation of differentiation, proliferation, metastasis, invasiveness, migration, and motility of malignant cells (Chaturvedi et al., 2007 ▶; Singh et al., 2004 ▶). The results showed that TQ has cytotoxic effects against pancreatic cancer cell line FG/COLO357 and down-regulated MUC4 expression through JNK and p38 MAPK pathways in a dose (0–100 µmol/L) and time-dependent manner (Torres et al., 2010 ▶).

Also, another study reported that pretreatment of pancreatic cancer cells with TQ (25 Mmol/L) for 48 h followed by gemcitabine or oxaliplatin, reduced growth of cancer cells (Banerjee et al., 2009 ▶).

 The effect of TQ (4 mg/kg/day) on diethylnitrosamine -induced hepatic carcinogenesis in rats was also studied. Findings documented that TQ could inhibit the development of DENA-induced liver cancer via decreasing oxidative stress and preserving the activity and expression of antioxidant enzymes (Sayed-Ahmed et al., 2010 ▶).

The anti-cancer effects of *N. sativa* and TQ were summarized in Table 1.

**Table 1:** Anti cancer effect of *N. sativa* and thymoquinone in GI tract

**Plant preparation**	**Experimental model**		**Effect**	**Reference**
***N. Sativa oil***		Colon cancer aberrant crypt foci were induced using1,2-dimethylhydrazine	Reduced total number of aberrant crypt foci	(Salim and Fukushima, 2003)
**Thymoquinone**		HCT-116 human colorectal cancer cells	Triggered apoptosis via a p53-dependent mechanism	(Gali-Muhtasib et al., 2004)
**Thymoquinone**		Pancreatic cancer cells	Cytotoxicity of pancreatic cancer cell line FG/COLO357M Down regulated MUC4 expression through JNK and p38 MAPK pathways	(Torres et al., 2010)
**Thymoquinone**		Pancreatic cancer cells	Reduced growth of cancer cells	(Banerjee et al., 2009)
**Thymoquinone**		Diethylnitrosamine induced hepatic carcinogenesis	Reduced oxidative stress Preserved the activity and expression of antioxidant enzymes	(Sayed-Ahmed et al., 2010)

**Hepatoprotective effect**

 The protective effect of *N. sativa* (0.2 mL/kg, intraperitoneal: i.p.) against hepatic ischemia/reperfusion injury was investigated in rats. Levels of serum aspartate aminotransferase (AST), alanine aminotransferase (ALT), and lactate dehydrogenase (LDH), total antioxidant capacity (TAC), catalase (CAT), total oxidative status (TOS), oxidative stress index (OSI) and myeloperoxidase (MPO) were measured. The results showed that *N. sativa* has a potential effect against hepatic ischemia/reperfusion injury and could act as a potent antioxidant agent (Yildiz et al., 2008 ▶).

 In another study, the effects of *N. sativa *(0.2 mL/ kg, i.p.) on cholestatic liver injury were evaluated in rats. The authors found that *N. sativa *has a preventing effect on cholestatic liver injury in rats. The results also suggested that the reduction of neutrophil infiltration and oxidative stress in the liver was probably responsible for this protective effect (Coban et al., 2010 ▶).

 In addition, the protective effect of *N. sativa* seeds (5% of the diet weight) against lead acetate-induced liver toxicity was documented in male rats. *N. sativa* seeds caused significant elevation in AST, improved biochemical and histopathological profiles and reduced damage areas (Farrag et al., 2007 ▶).

 The protective effects of *N. sativa* oil (0. 2 mL/kg, i.p.) and *Urticadioica* oil (2 mL/kg, i.p.) on carbon tetrachloride (CCl4)-induced liver toxicity were studied in rats. Findings showed that *N. sativa *and *U. dioica* reduced lipid peroxidation and liver enzymes, and enhanced antioxidant defense system activity in CCl4-treated rats (Kanter et al., 2005a ▶).

 The effect of TQ (10 mg/kg, orally) on hepato-renal dysfunction, CYP3A1 and spermidine/spermine N-1-acetyl-transferase gene expression induced by renal ischaemia/reperfusion was evaluated in rats. According to this study, TQ has a protective action on renal ischaemia/reperfusion-induced damage via an antioxidant mechanism and could decrease CYP3A1 and SSAT gene expression (Awad et al., 2011 ▶).

 The protective effect of TQ against tert-butyl hydroperoxide toxicity was evaluated in isolated rat hepatocytes. The results showed that pre-treatment of hepatocytes with 1 mM TQ reduced the leakage of cytosolic enzymes, ALT and AST (Daba and Abdel-Rahman, 1998 ▶).

 Oral administration of a single dose (100 mg/Kg) of TQ to male Swiss albino mice resulted in a protective effect against CCl4-induced hepatotoxicity which was probably due to the antioxidant property of TQ (Nagi et al., 1999 ▶).

 In another study, the protective effect of TQ (4.5, 9 and 18 mg/kg, i.p.) on Aflatoxin B1 -induced liver toxicity was evaluated in mice. Findings of this study showed that TQ significantly decreased AST, ALT, ALP and MDA levels. This protective effect may be mediated through increased resistance to oxidative stress as well as reduction in lipid peroxidation (Nili-Ahmadabadi et al., 2011 ▶).

 Thymoquinone showed protective effects against lipopolysaccharide -induced endotoxemia due to its anti-inflammatory, anti-apoptotic and antioxidant activities (Helal, 2010 ▶).

TQ (10 mg/kg, oral) protective effect on sodium ﬂuoride-induced hepatotoxicity and oxidative stress in rats was shown as it improved the antioxidant status and reduced the alterations in biochemical parameters. This protective effect was perhaps due to the ability of TQ to antagonize increased lipid peroxidation (LPO) and in turn stabilizing the integrity of the cellular membranes and decreasing the leakage of liver enzymes (Abdel-Wahab, 2013 ▶). Also, it was shown that TQ (50 mg/kg body weight) significantly inhibited tamoxifen-induced hepatic glutathione depletion and normalized the activity of SOD (Suddek, 2014 ▶).

 TQ (0.5, 1 and 2mg/kg/day, oral) combated against acetaminophen-induced hepatotoxicity and decreased acetaminophen-induced hepatotoxicity in a dose-dependent manner as evidenced by reduction in serum ALT activities. Hepatoprotective effect of TQ was probably mediated by increased resistance to oxidative and nitrosative stress and improved mitochondrial energy production (Nagi et al., 2010 ▶).

 In a clinical study, the effects of ethanolic extracts of *N. sativa*, *Zingiber officinale* (*Z. officinale*) and their mixture were evaluated in patients with hepatitis C virus (HCV) infection. Patients were divided into five groups: I) Healthy subjects, II) HCV control; III) HCV patients receiving a capsule containing 500 mg *N. sativa* extract twice daily; IV) HCV patients receiving a capsule containing 500 mg *Z. officinale* extract twice daily and V) HCV patients receiving a capsule containing 500 mg *Z. officinale* and 500 mg *N. sativa *extracts twice daily. The results showed that ethanolic extracts of *N. sativa* and *Z. officinale* had a significant effect in HCV patients as shown by a decrease in viral load and restoration of liver functions (Adel et al., 2013 ▶).

The hepatoprotective effects of *N. sativa* and TQ were summarized in Table 2.

**Table 2:** Hepatoprotective effect of *N. sativa* and thymoquinone

**Plant preparation**	**Experimental model**	**Effect**	**Reference**
***N. Sativa ***	Hepatic ischemia–reperfusion injury	Reduced levels of liver enzymes Antioxidant activity	(Yildiz et al., 2008)
***N. Sativa***	Cholestatic liver injury	Reduced neutrophil infiltration Reduced oxidative stress	(Coban et al., 2010)
***N. sativa*** ** seed**	Lead acetate induced liver toxicity	Increased AST	(Farrag et al., 2007)
***N. sativa *** **oil**	Trinitrobenzenesulphonic acid (TNBS)-induced colitis	Increased CAT activity Decreased LDH activity, TNF-α, IL-1β, IL-6	(Emekli-Alturfan et al., 2011)
***N. sativa*** ** oil**	Carbon tetrachloride (CCl4) induced liver toxicity	Reduced lipid peroxidation and liver enzymes, Increased antioxidant defense system activity	(Kanter et al., 2005a)
**Ethanolic extracts **	Hepatitis C virus (HCV) infection	Decreased viral load	(Adel et al., 2013)
**Thymoquinone**	Tert-butyl hydroperoxide (TBHP) induced liver toxicity	Reduced leakage of cytosolic enzymes, ALT and AST	(Daba and Abdel-Rahman, 1998)
**Thymoquinone**	Carbon tetrachloride (CCl4) induced liver toxicity	Antioxidant properties	(Nagi et al., 1999)
**Thymoquinone**	Aflatoxin B1 (AFB1) induced liver toxicity	Reduced AST, ALT, ALP and MDA levels	(Nili-Ahmadabadi et al., 2011)
**Thymoquinone**	Sodium ﬂuoride-induced hepatotoxicity	Antagonize the increased LPO Reduced the leakage of liver enzymes	(Abdel-Wahab, 2013)
**Thymoquinone**	Tamoxifen induced liver toxicity	Inhibited glutathione depletion Normalized the activity of SOD	(Suddek, 2014)
**Thymoquinone**	Hepatorenal dysfunction induced by renal ischaemia-reperfusion	Reduced damage via an antioxidant mechanism Reduced of CYP3A1 and SSAT gene expression	(Awad et al., 2011)
**Thymoquinone**	Acetaminophen induced hepatotoxicity	Reduced in ALT activity	(Nagi et al., 2010)

**Anti-bacterial and anti-schistosomiasis effects**

 The effect of *N. sativa* seed (0%, 1%, 2% and 3% of diet) on performance, intestinal *Escherichia coli* (*E. coli*) colonization and jejunal morphology in laying hens was evaluated. The results showed that ileal *E. coli* numeration reduced with 1% *N. sativa*. However, the best intestinal health indices were obtained following administration of 2% *N. sativa *(Boka et al., 2014 ▶).

 The effect of TQ (10 mg/kg, i.p.) against bacterial translocation and inflammatory responses induced by mechanical intestinal obstruction was studied in rats. The results indicated that TQ decreased inflammatory cytokines, oxidative damage, bacterial translocation and improved intestinal barrier function in rats with intestinal obstruction (Kapan et al., 2012 ▶).

 In a clinical trial, the effect of *N. sativa *seed in comparison with a triple therapy including clarithromycin, amoxicillin, and omeprazole against *Helicobacter pylori* (*H. pylori*) was evaluated in patients with non-ulcer dyspepsia. Patients were randomly divided into four groups: I) Triple therapy; II) 1 g/day *N. sativa* + 40 mg omeprazole; III) 2 g/day *N. sativa* + 40 mg omeprazole and IV) 3 g/day *N. sativa* + 40 mg omeprazole for four weeks. The results indicated that 2 g/day *N. sativa *+ 40 mg omeprazole has the best therapeutic effect on *H. pylori* activity (Salem et al., 2010 ▶). 

 The antioxidant and anti-schistosomal effects of garlic aqueous extract (125 mg kg -1, i.p.) and *N. sativa* oil (0.2 mg kg, i.p.) in normal mice and *Schistosoma mansoni* (*S. mansoni*)-infected mice were studied. Hematological parameters and levels of MDA, GSH, LDH, AST, and ALT were assessed in the liver. The results revealed that garlic extract and *N. sativa* oil reversed most of the hematological and biochemical changes and markedly improved the antioxidant capacity of treated infected mice as compared to untreated infected mice (Shenawy et al., 2008 ▶). 

 The effect of oral administration of N. Sativa oil (2.5 and 5 ml/kg) alone or in combination with praziquantel on liver injury induced by *S. mansoni* was investigated in mice. The results showed that* N. Sativa *oil reduced the number of *S. mansoni* worms in the liver and decreased the total number of ova deposited in both the liver and intestine. When *N. Sativa *oil was administered in combination with praziquantel, the most prominent effect was a further reduction in the dead ova number more than that induced by praziquantel alone (Mahmoud et al., 2002 ▶).

 The schistosomicidal effect of *N. sativa* seed against *S. mansoni* miracidia, cercariae, and adult worms was evaluated. Findings showed that *N. sativa* seed had a strong biocidal effect against all stages of the parasite life and showed inhibitory effect on egg-laying in adult female worms. The results also indicated that *N. sativa* seed induced oxidative stress against adult worms which was determined by reduction in the activities of antioxidant enzymes (Mohamed et al., 2005 ▶).

The anti-bacterial and anti-schistosomiasis effects of *N. sativa* and TQ were summarized in Table 3.

**Table 3:** Anti-bacterial and anti-schistosomiasis effect of *N. sativa* and thymoquinone in GI tract

**Plant preparation**	**Experimental model**	**Effect**	**Reference**
***N. sativa*** ** seed**	Intestinal escherichia coli colonization	Reduced numeration of ileal E. coli	(Boka et al., 2014)
***N. sativa*** ** seed**	Schistosomamansoni infection	Biocidal effect in all stages of the parasite Inhibitory effect on egg-laying of adult female worms	(Mohamed et al., 2005)
***N. sativa*** ** seed**	Patients with H. pylori	Potential effect on H. pylori activity	(Salem et al., 2010)
***N. sativa *** **oil**	Schistosomamansoni infection	Reduced the number of S. mansoni worms in the liver Reduced the total number of ova	(Mahmoud et al., 2002)
***N. sativa *** **oil**	Schistosomamansoni infection	Inhibited most of the hematological and biochemical changes Improved the antioxidant capacity	(Shenawy et al., 2008)
**Thymoquinone**	Bacterial translocation induced by mechanical intestinal obstruction	Reduced inflammatory cytokines, oxidative damage, bacterial translocation Improved intestinal barrier function	(Kapan et al., 2012)

**Anti-inflammatory and antioxidant effects**

 The effects of *N. sativa *oil (0.88 g/kg, orally) on gastric secretion and ethanol-induced ulcer in adult male rats were assayed. The results showed that *N. sativa *oil increased gastric mucin content, free acidity and glutathione level, and decreased gastric mucosal histamine content. It is concluded that *N. sativa *oil has a protective effect on ethanol-induced ulcer (El-Dakhakhny et al., 2000 ▶).

In another study, gastroprotective effects of *N. sativa *oil (2.5 and 5 ml/kg, orally) and TQ (5, 20, 50 and 100 mg/kg, orally) against gastric mucosal injury induced by ischaemia/reperfusion were evaluated in male Wistar rats. The results indicated that *N. sativa *oil and TQ at 5 and 20 mg/kg reduced LDH, LPO and increased GSH and SOD. It is concluded that *N. sativa *oil and TQ had a protective effect on gastric injury (El-Abhar et al., 2003 ▶).

 The effects of *N. sativa *oil (10 mL/kg body weight, orally) and TQ (10 mg/kg body weight, orally) against acute alcohol-induced gastric mucosal injury were investigated in male albino rats. The findings showed that *N. sativa *oil caused a reduction in ulcer index and MDA level and promoted healing of gastric injury and SOD, GSH and GST levels. Likewise, TQ has a protective activity on gastric lesions but less than that of *N. sativa *(Kanter et al., 2005b ▶)

 The gastroprotective and anti-secretory effects of *N. sativa *seed powder (1.0, 1.5 and 2.0 g/kg, oral), aqueous and ethanolic extracts of *N. sativa *seed powder (2.0 g/kg, oral), and *N. sativa *ethanol-ethyl acetate fraction (2.0 g/kg, oral) were investigated in indomethacin-treated rats. The results showed that *N. sativa *seed powder decreased indomethacin-induced gastric lesions in a dose-dependent manner. Ethanolic extract of *N. sativa *significantly reduced gastric secretion volume, pH, acid-output and ulcer index, whereas aqueous extract only decreased gastric acid-output (Rifat-uz-Zaman and Khan, 2004 ▶).

 In another study, the protective effect of *N. sativa* oil (10 ml/kg body weight) against piroxicam-induced gastric mucosal injury in adult male albino rats was investigated using light and scanning electron microscope. The results showed that *N .sativa* oil improved the structure of the mucosa in rats that received piroxicam and increased mucus secretion (Mohammed et al., 2010 ▶).

 The protective effect of *N. sativa *oil (10 ml/kg body weight) on stress-induced gastric ulcer in hypothyroid rats was studied. Animals were randomly divided into six groups: I) Control; II) Surgically thyroidectomized group; III) Acute cold restraint stressed group; IV) Surgically thyroidectomized and stressed group; V) *N. sativa* oil group and VI) Surgically thyroidectomized and stressed receiving *N. sativa* oil group. Findings indicated a reduction in thyroid hormone level and an increase in stress-induced gastritis which can be inhibited by *N. sativa* oil (Abdel Sater, 2009 ▶).

The effects of two-week administration of *N. Sativa* oil (0.88 mL/kg/day, orally), omeprazole (30 mg/kg body weight/day, orally) and corn oil (2 mL/kg/day, orally) on ethanol-induced gastric lesions were studied in rats. The results indicated that *N. sativa* oil significantly increased glutathione and antioxidant enzymes and decreased lipid peroxides and protein carbonyl content. It is concluded that co-administration of omeprazole and *N. sativa* oil significantly improved all of the studied parameters (El-Masry et al., 2010 ▶).

 In one study, the effects of TQ (10 and 20mg/kg), omeprazole (10 and 20mg/kg) or co-administration of TQ (10mg/kg) and omeprazole (10mg/kg) on gastric mucosal ischemia/reperfusion injury induced by pyloric ligation (30 min), ischemia (30 min)/reperfusion (120 min) were investigated in rats. The results revealed that TQ had gastroprotective effects which were mediated by inhibiting proton pump, acid secretion and neutrophil infiltration, and increasing mucin secretion, and nitric oxide production (Magdy et al., 2012).

 The antioxidative and anti-histaminergic effects of *N. sativa *(500mg/kg, oral) and TQ (10mg/kg, orally) on ethanol-induced gastric mucosal damage were investigated in rat. The results showed that *N. sativa* signiﬁcantly decreased the number of mast cells, the area of gastric erosions, histamine levels and myeloperoxidase activity. However, TQ effect was less pronounced as compared to that of *N. sativa*. The results also suggested that gastroprotective effects of *N. sativa* could be due to its anti-peroxidative, anti-oxidant and anti-histaminergic effects (Kanter et al., 2006 ▶).

 The effect of *N. *seed oil (2.5 ml/kg, orally) on gastric tissues in experimental colitis (trinitrobenzenesulphonic acid -induced colitis) was studied. The levels of sialic acid (SA), GSH, MDA and CAT and SOD activities in gastric tissue samples and TNF-α, IL-1β and IL-6 and LDH levels in blood samples were determined. *N. sativa* seed oil significantly increased gastric tissue CAT activity and decreased LDH activity and TNF-α, IL-1β, IL-6 levels. Findings of this study indicated that *N. sativa* seed oil has a modulatory effect on inflammatory response in colitis (Emekli-Alturfan et al., 2011 ▶).

 The effect of TQ (5 and 10 mg/kg) and sulfasalazine (500 mg/kg) as an anticolitis drug on acetic acid-induced colitis (by intracolonic injection of 3% acetic acid) was investigated in rats. Findings revealed that TQ has a more pronounced protective effect on colitis as compared to sulfasalazine and this effect may be possibly mediated through its antioxidant action (Mahgoub, 2003 ▶).

 The effect of TQ (100 mg/kg, orally) on chronic pancretitis induced by high fat diet and ethanol was studied in rats. Findings revealed that TQ has a protective effect on pancretitis via reducing the secretion of amylase and lipase from pancreas, inflammatory cytokine and lipid peroxidation (Suguna et al., 2013 ▶).

Various gastroprotective, anti-inflammatory and anti-oxidant effects of *N. sativa* and TQ were summarized in Table 4.

**Table 4:** Anti-inflammatory and antioxidant effect of *N. sativa* and thymoquinone in GI tract

**Plant preparation**	**Experimental model**	**Effect**	**Reference**
***N. Sativa***	Ethanol induced gastric mucosal damage	Reduced the number of MC , the area of gastric erosions, histamine levels and myeloperoxidase activity	(Kanter et al., 2006)
***N. sativa *** **seed**	Gastric lesions induced by indomethacin	Reduced the gastric lesions	(Rifat-uz-Zaman and Khan, 2004)
**Aqueous extract **	Gastric lesions induced by indomethacin	Reduced gastric acid-output	(Rifat-uz-Zaman and Khan, 2004)
**Ethanolic extract **	Gastric lesions induced by indomethacin	Reduced gastric secretion volume, pH, acid-output and ulcer index.	(Rifat-uz-Zaman and Khan, 2004)
**ethanol ethyl acetate 51 fractions**	Gastric lesions induced by indomethacin	Potential effect on pepsin activity, ulcer index and gastric secretion	(Rifat-uz-Zaman and Khan, 2004)
***N. Sativa*** ** oil**	Gastric mucosal injury induced by ischaemia reperfusion	Reduced LDH, LPX Increased GSH, SOD	(El-Abhar et al., 2003)
***N. Sativa*** ** oil**	Alcohol-induced gastric mucosal injury	Reduced in the ulcer index, MDA Promoted healing of gastric injury, SOD, GSH, GST.	(Kanter et al., 2005b)
***N. Sativa*** ** oil**	Piroxicam induced gastric mucosal injury	Improved the structure of the mucosa Increased in mucus secretion	(Mohammed et al., 2010)
***N. Sativa*** ** oil**	Stress gastriculcer in hypothyroidal rats	Reduced in thyroid hormone level increased stress gastritis, and this effect can be inhibited by treatment with *N. sativa* oil	(Abdel Sater, 2009)
***N. Sativa*** ** oil**	Trinitrobenzene sulfonic acid (TNBS)induced experimental colitis	Reduced the proinﬂammatory cytokines, lactate dehydrogenase, myeloperoxidase, triglyceride, cholesterol and increasd superoxide dismutase activity.	(Isik et al., 2011)
***N. Sativa*** ** oil**	Ethanol induced gastric lesions	Increased glutathione and antioxidant enzymes Reduced lipid peroxides and protein carbonyl content	(El-Masry et al., 2010)
***N. Sativa*** ** oil**	Ethanol induced ulcer	Increased the gastric mucin, free acidity and glutathione level Reduced the gastric mucosal histamine content	(El-Dakhakhny et al., 2000)
**Thymoquinone**	Acetic acid-induced colitis	Antioxidant activity	(Mahgoub, 2003)
**Thymoquinone**	Gastric mucosal ischemia/reperfusion (I/R) injury	Inhibited proton pump, acid secretion and neutrophil infiltration Increased mucin secretion, and nitric oxide production	(Magdy et al., 2012)
**Thymoquinone**	Chronic pancretitis induced by high fat diet and ethanol	Reduced the secretion of amylase and lipase from pancreas, inflammatory cytokine and lipid peroxidation	(Suguna et al., 2013)

## Conclusion

*N. sativa* and its main constituent, TQ showed anti-cancer, hepatoprotective, anti-bacterial, anti-schistosomiasis, gastroprotective, anti-inflammatory and antioxidant effects in animal models of gastrointestinal disorders including cancers, hepatotoxicity, ischemia/reperfusion injury, cholestatic liver, non-ulcer dyspepsia, schistosomiasis infection, colitis and panceratitis. These effects supported the preventive and therapeutic effect of *N. sativa* and its constituents on inflammatory, oxidative and toxic injury due to various toxins, microbes and food allergens. Clinical studies also indicated preventive effect as well as relieving effect of this plant and its constituents on various gastrointestinal disorders. Therefore, *N. sativa* have both preventive and therapeutic effects on gastro-intestinal diseases.

However, further clinical and experimental investigations are required to reveal the exact perspective of molecular and cellular basis of the effects of *N. sativa* and its constituents. 
